# Neutrophil‐to‐Lymphocyte Ratio Predicts Dialysis Timing & Prognosis in Critically Ill Patients

**DOI:** 10.1002/hsr2.70313

**Published:** 2025-02-03

**Authors:** Abdulqadir J. Nashwan, Mutaz I. Othman, Dore C. Ananthegowda, Kalpana Singh, Anas Ibraheem, Jayesh P. Janardhanan, Jamsheer P. Alikutty, Muftah A. Othman, Abdullah I. Hamad, Mohamad Y. Khatib, Ahmad A. Abujaber

**Affiliations:** ^1^ Nursing Department Hamad Medical Corporation Doha Qatar; ^2^ Critical Care Department Hamad Medical Corporation Doha Qatar; ^3^ Hematology Department Al Karama Teaching Hospital Baghdad Iraq; ^4^ Nephrology Department Hamad Medical Corporation Doha Qatar

**Keywords:** acute kidney injury (AKI), continuous renal replacement therapy (CRRT), intensive care unit (ICU), morbidity, mortality, neutrophil‐to‐lymphocyte ratio (NLR)

## Abstract

**Background and Aims:**

The neutrophil‐to‐lymphocyte ratio (NLR) is a cost‐effective indicator of inflammation, which may impact decisions regarding therapy for patients undergoing continuous renal replacement therapy (CRRT), even with ongoing clinical arguments. This study aimed to examine the correlation between NLR and the prognosis of critically ill patients undergoing CRRT, specifically about mortality and morbidity. Additionally, the study sought to assess NLR's potential as a prognostic indicator for CRRT initiation.

**Methods:**

Data were retrospectively analyzed from 175 critically ill patients who received CRRT. Clinical factors and biochemical markers were compared between survivors and non‐survivors at admission, before CRRT, and at 24 and 72 h post‐CRRT initiation.

**Results:**

Elevated NLR levels were significantly associated with increased in‐hospital mortality. Neutrophil counts showed statistical significance across all measurement points, while NLR and lymphocyte counts were significant only on the third day of CRRT (*p* 0.001 and 0.011, respectively). Non‐survivors had higher NLR values than survivors and experienced shorter hospital stays (median 22 vs. 44 days for survivors, *p* < 0.001). Patients with higher baseline NLR values also had more complications.

**Conclusions:**

The NLR shows potential as a prognostic predictor for mortality in CRRT patients. Its integration into clinical practice could enhance patient care and treatment timing, and further studies should validate its clinical utility.

## 1 Introduction

Acute kidney injury (AKI) is a significant predictor of adverse patient outcomes [1]. AKI affects around 13.3 million patients annually, with 85% of cases occurring in developing countries. While a direct causal relationship between AKI and mortality has not been shown, it is believed that AKI contributes to approximately 1.7 million deaths each year [2]. Continuous renal replacement therapy (CRRT) is a frequently employed renal support modality for the management of critically ill patients experiencing AKI, specifically for those who are experiencing hemodynamic instability. CRRT typically lasts up to 24 h when admitted in an intensive care setting [3]. CRRT is a method for maintaining solute and fluid balance through slower, continuous dialysis, with various techniques varying in solute removal approach, primarily influenced by physician preference [4]. This differentiates it from standard renal replacement therapies such as intermittent hemodialysis, which usually span 4−6 h or less [5].

The neutrophil‐to‐lymphocyte ratio (NLR), an inexpensive approach to measuring inflammation, could significantly affect the course of treatment for people on CRRT. NLR is a crucial parameter for evaluating inflammation, predicting and diagnosing disorders, determining mortality in cardiac events, and assessing the prognosis of various malignancies [6, 7]. It is a distinctive parameter that combines two fundamental components of the immune system, namely the innate and adaptive immune responses. In recent years, extensive research has been conducted to evaluate the effectiveness of this approach for patients with various medical disorders [8, 9]. The findings have demonstrated that it can be used in these patients. While there is an ongoing argument on the exact cutoff value of NLR, its significance as a biomarker for maintaining immune system balance has been well established [10].

The prognosis of CRRT patients is determined by various factors, such as age, body mass index, Acute Physiology and Chronic Health Evaluation II (APACHE II) and Sequential Organ Failure Assessment (SOFA) scores, systolic and diastolic blood pressure, and biochemical markers, including serum creatinine and serum sodium levels [11]. Despite advancements in healthcare nowadays, the mortality rate among these patients remains significantly elevated, surpassing the threshold of 50% [12, 13].

Several scoring systems, such as the APACHE II score, SOFA score, and Simplified Acute Physiology Score (SAPS), have been utilized in previous studies to forecast the clinical outcomes of critically ill patients receiving CRRT [14]. However, there remains a lack of a reliable and autonomous prognostic factor that is cost‐effective for these patients. Especially in developing countries, the comprehensibility of accepted scoring systems might be challenging because of limited access to studies and resources [15]. Furthermore, despite the numerous advancements in clinical practice, there is significant speculation surrounding the optimal period for starting CRRT.

Although several large‐scale studies have been conducted over the past few years to determine the optimal timing for initiating CRRT, a definitive consensus on the most advantageous time has yet to be reached. The ELAIN trial, a randomized controlled trial conducted in a single center in Germany, examined the impact of early versus delayed initiation of renal replacement therapy on mortality in critically ill patients with AKI. The trial findings indicated that initiating therapy earlier resulted in a more favorable mortality outcome within 90 days [16]. In contrast, the Artificial Kidney Initiation in Kidney Injury (AKIKI) trial and the Initiation of Dialysis Early Versus Delayed in the Intensive Care Unit (IDEAL‐ICU) trial, both multicenter randomized controlled trials conducted in France, did not find any differences in the overall outcome between early and late initiation of treatment [17].

This study aims to investigate the correlation between the NLR and the prognosis of critically ill patients on CRRT, specifically regarding mortality and morbidity outcomes. Furthermore, it aims to investigate the efficacy of NLR as a potential indicator for the optimal time for CRRT starting points.

## 2 Methods

### 2.1 Study Oversight

The Institutional Review Board (IRB) of the Medical Research Committee (MRC) at Hamad Medical Corporation (HMC) officially approved our retrospective cohort study, identified as MRC‐01‐23‐755. Following a systematic procedure, we proceeded with data collection. The first step was identifying the target population and extracting relevant data from medical records and other sources in the database, the collected data were anonymous. The present study focuses on the impact of the NLR on outcomes, particularly at admission and after 24, 48, and 72 h of initiating CRRT, as well as dependent variables, which include mortality and morbidity outcomes. This could consist of variables such as the length of hospital or ICU stay, the frequency of complications, or specific clinical outcomes for critically ill patients. Also consider the relationship between NLR levels and the timing of CRRT initiation as an outcome to determine whether NLR levels can help predict when CRRT should begin for optimal patient outcomes.

The data underwent a very careful cleaning and validation process to ensure accuracy. The data was stored securely and analyzed using appropriate statistical methods to meet the research objectives. The research findings have been carefully analyzed and synthesized into a comprehensive report that adheres to ethical guidelines and regulations in accordance with the principles stated in the Declaration of Helsinki, prioritizing the protection of patient privacy and data integrity.

### 2.2 Study Design

A retrospective cohort study was conducted to collect baseline patients' characteristics, laboratory findings, CRRT complications, and in‐hospital courses. The information was retrieved from electronic medical records (EMR), allowing for a comprehensive analysis of patients' data.

### 2.3 Population

The study used convenience sampling to collect data from April 2020 to 2023 at HMC intensive care units (ICU). It included all critically ill patients aged 18 years and above who were admitted to the ICU and received CRRT. It excluded critically ill patients with incomplete data or those receiving CRRT for end‐of‐life care or end‐stage malignancy. The sample size of 175 patients included in this retrospective study was derived from a comprehensive review of medical records within a specified time frame. The selection process aimed to include all eligible patients who met the inclusion criteria, which were defined based on specific criteria such as diagnosis and treatment received.

### 2.4 Data Analysis

The collected data were summarized using appropriate descriptive statistics, including frequencies and percentages for categorical variables (e.g., gender, CRRT indications and complications, and patient's clinical progression) and means with standard deviations (SD) or medians for continuous variables (e.g., age, length of hospitalization, and SOFA score). To assess associations between mortality and variables, such as demographic factors, CRRT indications and complications, and length of hospitalization, bivariate analyses were conducted. Specifically, independent *t*‐tests or Mann−Whitney *U* tests were applied for continuous variables based on their distribution, while Chi‐square or Fisher's exact tests were used for categorical variables. Logistic regression analysis was used to investigate the associations between survival status and variables, including sociodemographic factors, CRRT complications, length of hospitalization, and NLR at the time of admission, second day, and third day. All statistical analyses were performed using STATA version 17, with a significance level set at a *p*‐value of < 0.05 (two‐tailed test).

A thorough investigation into morbidity regarding CRRT complications and hospitalization duration was conducted through bivariate analyses, employing independent *t*‐tests or Mann−Whitney *U* tests for continuous variables. These analyses explored their relationship with baseline NLR before CRRT initiation. Additionally, the predictive potential of NLR for mortality was evaluated using similar bivariate analyses. These assessments encompassed all laboratory parameters at admission, pre‐CRRT initiation, and 24 and 72 h post‐CRRT commencement. To assess the favorable initiation time for CRRT, the outcomes of the included patients were tested retrospectively against the baseline NLR findings at the time of admission. These findings were analyzed using appropriate statistical tests, depending on the distribution and nature of the data. For example, chi‐square or Fisher's exact tests were used to compare categorical outcomes.

### 2.5 NLR Calculation and Cut‐Off Point

NLR was measured by dividing the number of neutrophils by the number of lymphocytes from the same venous blood sample collected at admission, before CRRT initiation, and subsequent endpoints at 24 and 72 h after CRRT initiation [1]. The cut‐off point distinguishing high and low NLR is set at 3, with an NLR of 3 or greater considered high.

### 2.6 SOFA Score Calculation

The SOFA score is crucial for defining patient clinical conditions and therapy responses. It comprises six distinct scores, one for each of the respiratory, cardiovascular, hepatic, coagulation, renal, and central nervous systems, which make up the result for each organ system. Each receives a score ranging from 0 (normal) to 4 (most abnormal), with a minimum SOFA score of 0 and a maximum SOFA score of 24 [18].

## 3 Results

Out of 197 patients, 22 patients were excluded, 21 critically ill patients with incomplete data, and 1 patient receiving CRRT for end‐of‐life care or end‐stage malignancy. The study included a total of 175 patients who met inclusion criteria, comprising 18 survivors and 157 deceased individuals, resulting in an overall mortality rate of 89.7%. Table 1 summarizes the patients' baseline characteristics. The cohort had a mean age of 60.9 ± 16.0 years, with a majority of 81.7% being male and the remaining 18.3% being female. The median hospital stay was 25.00 days, with an interquartile range [IQR] of 14.00−41.00 days. A high percentage of the subjects had a history of chronic kidney disease (CKD), with a significant number also having a history of diabetes. In addition, most patients (98.3%) were admitted to the hospital for 3 days or longer. Upon admission, patients were presented with various indications for CRRT, including metabolic acidosis, hyperkalemia, hyponatremia, hyperphosphatemia, uremic encephalopathy, and persistent or progressive acute renal failure. As well as complications of CRRT, such as hemorrhage, infection, venous thrombosis, pneumothorax, haemothorax, circuit thrombosis, electrolyte disturbances, hemolysis, hypothermia, and hypotension, were observed in different percentages of cases.

**Table 1:** General profile and characteristics of patients (*n* = 175).

Variable			Total	Survivor	Non‐survivor	*p* value
			175 (100%)	18 (10.3%)	157 (89.7%)	
Age, mean (±SD)			60.87 (±15.94)	48.22 (±8.80)	62.32 (±15.95)	< 0.001*
Sex		Male	143 (81.7%)	17 (94.4%)	126 (80.3%)	0.14
		Female	32 (18.3%)	1 (5.6%)	31 (19.7%)	
History of chronic kidney disease		Yes	173 (98.9%)	17 (94.4%)	156 (99.4%)	0.063
		No	2 (1.1%)	1 (5.6%)	1 (0.6%)	
History of diabetes mellitus		Yes	75 (43.1%)	8 (44.4%)	67 (42.9%)	0.90
		No	99 (56.9%)	10 (55.6%)	89 (57.1%)	
Length of hospitalization, median (IQR) days			25.00 (14.00, 41.00)	44.00 (34.00, 88.0)	22.00 (13.00, 37.00)	< 0.001*
Length of hospitalization		< 3 days	3 (1.7%)	0 (0.0%)	3 (1.9%)	0.55
		≥ 3 days	172 (98.3%)	18 (100%)	154 (98.1%)	
Indication for CRRT	Volume overload	Yes	53 (30.3%)	5 (27.8%)	48 (30.6%)	0.81
		No	122 (69.7%)	13 (72.2%)	109 (69.4%)	
	Metabolic acidosis	Yes	101 (57.7%)	8 (44.4%)	93 (59.2%)	0.23
		No	74 (42.3%)	10 (55.6%)	64 (40.8%)	
	Hyperkalemia	Yes	63 (36.0%)	5 (27.8%)	58 (36.9%)	0.44
		No	112 (64.0%)	13 (72.2%)	99 (63.1%)	
	Hyponatremia	Yes	8 (4.6%)	2 (11.1%)	6 (3.8%)	0.16
		No	167 (95.4%)	16 (88.9%)	151 (96.2%)	
	Hyperphosphatemia	Yes	37 (21.1%)	2 (11.1%)	35 (22.3%)	0.27
		No	138 (78.9%)	16 (88.9%)	122 (77.7%)	
	Uremic encephalopathy	Yes	29 (16.6%)	4 (22.2%)	25 (15.9%)	0.50
		No	146 (83.4%)	14 (77.8%)	132 (84.1%)	
	Uremic pericarditis	Yes	3 (1.7%)	0 (0.0%)	3 (1.9%)	0.55
		No	172 (98.3%)	18 (100.0%)	154 (98.1%)	
	Persistent/progressive acute renal failure	Yes	135 (77.1%)	16 (88.9%)	119 (75.8%)	0.21
		No	40 (22.9%)	2 (11.1%)	38 (24.2%)	
Complications of CRRT	Hemorrhage	Yes	13 (7.4%)	0 (0.0%)	13 (8.3%)	0.20
		No	162 (92.6%)	18 (100.0%)	144 (91.7%)	
	Infection	Yes	6 (3.4%)	0 (0.0%)	6 (3.8%)	0.40
		No	169 (96.6%)	18 (100.0%)	151 (96.2%)	
	Venous thrombosis	Yes	4 (2.3%)	0 (0.0%)	4 (2.5%)	0.49
		No	171 (97.7%)	18 (100.0%)	153 (97.5%)	
	Pneumothorax	Yes	1 (0.6%)	1 (5.6%)	0 (0.0%)	< 0.01*
		No	174 (99.4%)	17 (94.4%)	157 (100.0%)	
	Hemothorax	Yes	1 (0.6%)	0 (0.0%)	1 (0.6%)	0.73
		No	174 (99.4%)	18 (100.0%)	156 (99.4%)	
	Air embolism	No	175 (100%)	18 (100.0%)	157 (100.0%)	
	Visceral injury	No	175 (100%)	18 (100.0%)	157 (100.0%)	
	Circuit thrombosis	Yes	35 (20.0%)	4 (22.2%)	31 (19.7%)	0.80
		No	140 (80.0%)	14 (77.8%)	126 (80.3%)	
	Electrolytes disturbance	Yes	7 (4.0%)	1 (5.6%)	6 (3.8%)	0.72
		No	168 (96.0%)	17 (94.4%)	151 (96.2%)	
	Hemolysis	Yes	2 (1.1%)	0 (0.0%)	2 (1.3%)	0.63
		No	173 (98.9%)	18 (100.0%)	155 (98.7%)	
	Allergic reaction to hemodialyzer/hemofilter/tubing	No	175 (100%)	18 (100.0%)	157 (100.0%)	
	Hypothermia	Yes	30 (17.1%)	0 (0.0%)	30 (19.1%)	< 0.05*
		No	145 (82.9%)	18 (100.0%)	127 (80.9%)	
	Hypotension	Yes	105 (60.0%)	5 (27.8%)	100 (63.7%)	< 0.01*
		No	70 (40.0%)	13 (72.2%)	57 (36.3%)	

*Note:* Test statistics: Chi‐square test for categorical variables and *t*‐test and Mann−Whitney *U* test for continuous variables. A *p*‐value less than 0.05 is considered statistically significant. Values marked with an asterisk (*) indicate a statistically significant difference between survivors and non‐survivors.

Abbreviations: CRRT, continuous renal replacement therapy; NLR, neutrophil‐to‐lymphocyte ratio; SOFA, sequential organ failure assessment.

Table 1 presents a comparative analysis between survivors and non‐survivors among the patients. A statistically significant age difference was observed between surviving patients (mean age: 48.22 ± 8.80 years) and deceased patients (mean age: 62.32 ± 15.95 years) (*p* < 0.001). Furthermore, deceased patients had a significantly shorter duration of hospitalization (22.00 days, IQR: 13.00−37.00) compared to surviving patients (44.00 days, IQR: 34.00−88.00) (*p* < 0.001). While no statistically significant differences were observed between the survival and non‐survival groups concerning CRRT indications, notable disparities emerged regarding its complications. Specifically, occurrences of pneumothorax, hypothermia, and hypotension were found to be statistically significant.

Regarding morbidity, as outlined in Table 2, no significant differences were observed in overall CRRT complications and length of hospitalization in association with the baseline NLR, where both *p*‐values were higher than 0.05.

**Table 2:** Utility of NLR in predicting morbidity associated with complications of CRRT and length of hospitalization by Mann−Whitney test results (*n* = 175).

Variable	Overall	Morbidity		*p* value
		Non‐ complicated CRRT	Complicated CRRT	
NLR at the time of admission, median (IQR)	19.76 (11.16, 36.42)	21.985 (11.83, 39.97)	18.06 (11.17, 35.29)	0.439
		Length of hospitalization < 3 days	Length of hospitalization ≥ 3 days	
	20.33 (11.61, 37.32)	56.29 (35.41, 57.99)	19.91 (11.62, 37)	0.260

*Note:* Test statistics: Mann−Whitney *U* test. A *p*‐value less than 0.05 is considered statistically significant.

Regarding mortality, the neutrophil count exhibited statistical significance across all three endpoints, while the NLR and lymphocyte count showed statistical significance only on the third day of CRRT, with *p*‐values of 0.001 and 0.011, respectively, as summarized in Table 3. Additionally, the SOFA score at the time of admission was not associated statistically with mortality (*p* = 0.47).

**Table 3:** Predictive value of NLR and SOFA score for mortality in CRRT patients (*n* = 175).

Variable	Overall	Mortality		*p* value
		Survivors	Non‐survivors	
SOFA score at the time of admission, median (IQR)	13.00 (11.00, 16.00)	13.00 (10.00, 15.00)	13.00 (11.00, 16.00)	0.47
Neutrophil at the time of admission, median (IQR)	90.60 (85.70, 93.90)	87.45 (84.00, 91.30)	90.70 (86.00, 94.10)	< 0.05*
Neutrophil Day 1, median (IQR)	91.00 (84.20, 94.40)	85.00 (79.40, 87.60)	91.70 (87.00, 95.00)	< 0.01*
Neutrophil Day 3, median (IQR)	87.20 (74.30, 93.30)	84.00 (73.70, 86.90)	88.95 (79.25, 94.45)	< 0.05*
Lymphocyte at the time of admission, median (IQR)	4.50 (2.50, 7.40)	5.10 (3.90, 7.10)	4.40 (2.50, 7.40)	0.33
Lymphocyte Day 1, median (IQR)	4.20 (2.10, 7.60)	6.00 (2.40, 9.00)	3.40 (2.10, 6.70)	0.18
Lymphocyte Day 3, median (IQR)	4.35 (2.30, 8.70)	8.45 (4.20, 9.20)	3.90 (2.00, 7.70)	< 0.05*
NLR at the time of admission, median (IQR)	20.33 (11.62, 37.32)	17.65 (11.17, 24.36)	21.07 (11.92, 38.12)	0.26
NLR Day 1, median (IQR)	12.57 (12.57, 27.29)	11.18 (6.62, 39.21)	12.57 (12.57, 27.09)	0.20
NLR Day 3, median (IQR)	11.99 (11.99, 11.99)	9.42 (5.54, 19.75)	11.99 (11.99, 11.99)	< 0.01*

*Note:* Test statistics: Mann−Whitney *U* test. A *p*‐value less than 0.05 is considered statistically significant. Values marked with an asterisk (*) indicate a statistically significant difference between survivors and non‐survivors.

Abbreviations: CRRT, continuous renal replacement therapy; NLR, neutrophil‐to‐lymphocyte ratio; SOFA, sequential organ failure assessment.

**Figure 1:**
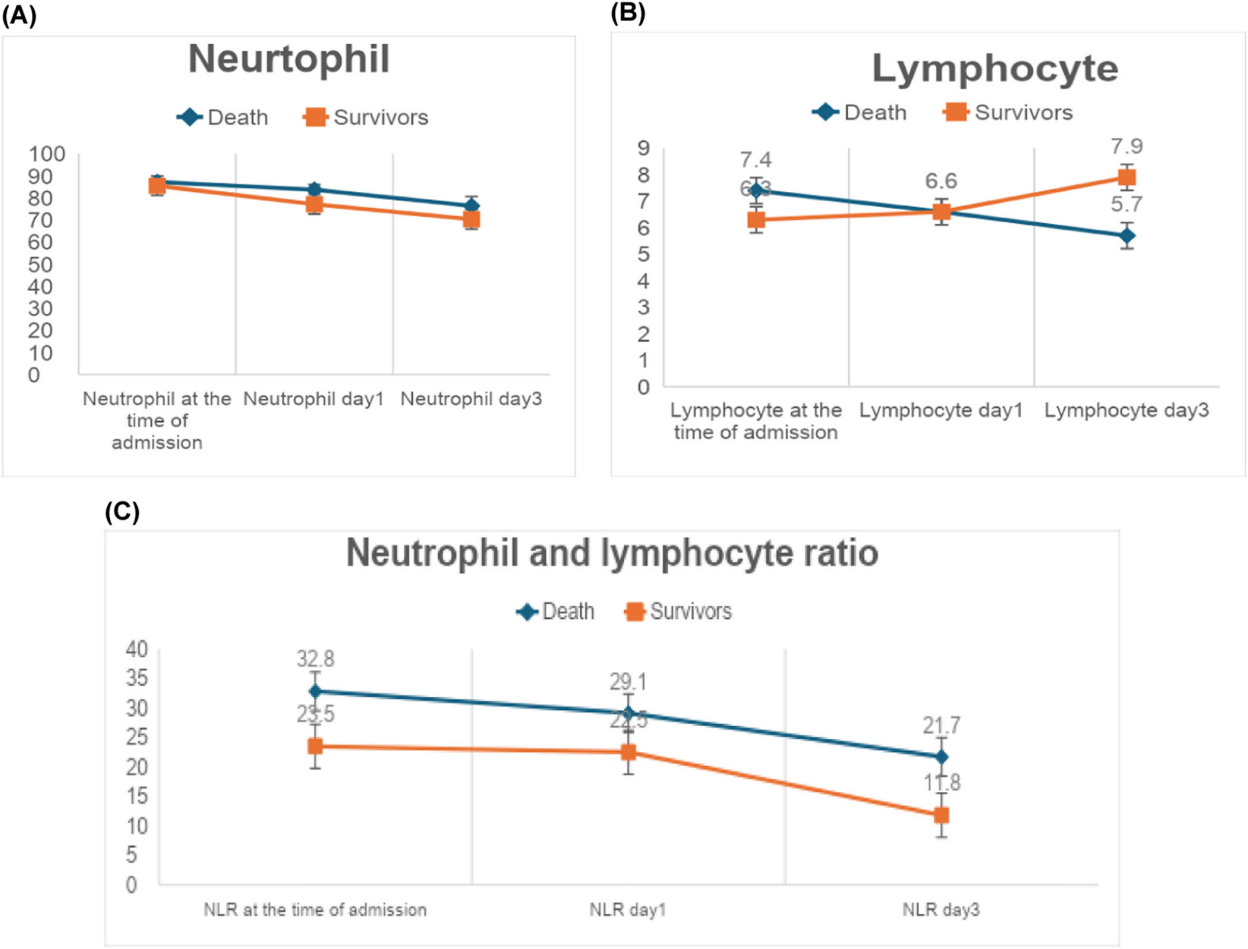
Dynamic changes of neutrophil (A), lymphocyte (B), and neutrophil‐to‐lymphocyte ratio (NLR) (C) during the first 3 days.

Figure 1 represents the dynamic changes of neutrophils and lymphocytes following the commencement of CRRT. Consequently, there was a significant variance in neutrophil and lymphocyte counts between survivors and non‐survivors.

Table 4 presents the crude and adjusted odds ratios (OR) with 95% confidence intervals (CI) for factors associated with mortality among patients undergoing CRRT. Age, length of hospital stays (LOS), sodium levels, and hypotension were significant predictors of mortality in both crude and adjusted models. Specifically, older age was associated with higher mortality, each 1‐year increase in age was associated with a 5% higher odds of mortality (adjusted OR: 1.05, 95% CI: 1.01−1.1, *p* = 0.020), while a longer hospital stay was associated with reduced mortality, with each additional day decreasing the odds of death by 3% (adjusted OR: 0.97, 95% CI: 0.95−0.99, *p* = 0.001). Sodium levels were also a significant predictor, with each unit increase associated with 11% higher odds of mortality (adjusted OR: 1.11, 95% CI: 1.00−1.23, *p* = 0.047), and hypotension emerged as a strong predictor of mortality, with patients experiencing hypotension having 4.56 times higher odds of death compared to those without hypotension (adjusted OR: 4.56, 95% CI: 1.22−17.03, *p* = 0.024). In contrast, phosphorus and urea levels, along with SOFA score and NLR values at admission on Day 1 and Day 3, were not significantly associated with mortality in the adjusted model.

**Table 4:** Crude and adjusted odds ratios for factors associated with mortality in patients undergoing CRRT.

Factor	Level	Crude OR 95% (CI)^a^	*p* value	Adjusted OR 95% (CI)^b^	*p* value
Age		1.06 (1.02, 1.09)	0.001	1.05 (1.01, 1.1)	0.020
Length of stay in days		0.97 (0.96, 0.99)	< 0.001	0.97 (0.95, 0.99)	0.001
Hypotension	No	Ref		Ref	
	Yes	4.56 (1.55, 13.45)	< 0.01	4.56 (1.22, 17.03)	0.024
Sofa score		1.08 (0.93, 1.27)	0.306	—	—
NLR at the time of admission		1.01 (0.99, 1.03)	0.283	—	—
NLR Day 1		1.00 (0.99, 1.02)	0.700	—	—
NLR Day 3		1.04 (0.97, 1.11)	0.249	—	—

^a^ Model: Unadjusted model for age, length of stay, sodium, phosphorus, urea, hypotension, SOFA score, and NLR at the time of admission, second day and third day.

^b^ Model: Adjusted model for age, length of stay, sodium, phosphorus, urea, and hypotension.

## 4 Discussion

This study aimed to investigate the association between the NLR and the prognosis of critically ill patients undergoing CRRT, specifically focusing on mortality and morbidity outcomes. The study assessed the effectiveness of NLR as a potential marker for determining the ideal timing to start CRRT. The key findings of our study provide valuable insights into the predictive usefulness of NLR for CRRT patients. The study found significant associations between NLR levels and in‐hospital mortality, consistent with prior research [9, 19]. The non‐survivors exhibited higher NLR values than the survivors, suggesting that NLR may serve as a predictive biomarker in this population. A study conducted in China investigated the utility of NLR as a biomarker to predict mortality in AKI cases. The study found that a higher level of NLR was associated with an increased risk of 30‐ and 90‐day mortality in AKI patients. However, this trend was not observed in the analysis of in‐hospital mortality [20].

Another study by Chen et al. investigated the usefulness of NLR in predicting the progression of AKI. The study found that NLR level at the time of AKI diagnosis was associated with adverse outcomes, including AKI progression, the requirement of renal replacement therapy, or in‐hospital mortality. This study demonstrated that NLR serves as an independent indicator of both AKI progression and in‐hospital mortality. The NLR's impact on the primary outcome was most significant when the NLR ranged from 7 to 38, as indicated by the lowest OR. Given its widespread availability in daily practice, this could be a valuable tool for risk stratification within the AKI population [21]. These rationales strongly support our study findings, indicating that the NLR holds promise as an alternative indicator for disease severity and prognosis in critically ill patients undergoing CRRT. By reflecting the systemic inflammatory response, NLR offers valuable insights into patient outcomes. Notably, the SOFA score, a widely acknowledged tool for assessing clinical outcomes in critically ill patients on CRRT [14], was statistically insignificant at admission, similar to the NLR. This suggests that the SOFA score may not possess a superior predictive ability compared to NLR in assessing the prognoses of CRRT patients. However, it is essential to acknowledge that this comparison was not the focus of our study, and further investigation is warranted to explore this matter thoroughly.

An NLR > 3 is associated with a higher risk of cardiovascular events, infection, and other complications associated with CRRT, which can ultimately result in a worse prognosis. The NLR is a robust prognostic indicator for assessing disease severity and mortality [22]. Given the absence of a universally recognized NLR cutoff value applicable to all clinical scenarios and the lack of prior literature discussing the CRRT−NLR relationship, calculating the optimal cutoff point using the receiver operating characteristic (ROC) curve would provide a tailored and evidence‐based interpretation of NLR's predictive ability in this specific context. Additionally, when analyzing changes in the NLR, it is important to consider various factors, such as nutritional status, underlying diseases, and treatment regimens. In clinical practice, physicians should treat patients with aberrant NLRs cautiously, monitor the NLR regularly, and take immediate action to reduce the risk of illness [23].

By utilizing CRP levels and the NLR, physicians can enhance their ability to predict patient complication risk and prognosis. This, in turn, allows for early intervention and management to minimize the chances of severe complications [24]. However, there are still some limitations associated with the data reported. These include the limited number of clinical studies reviewed, the relatively brief duration of follow‐up in most studies, insufficient data, and the variability of NLR in dialysis patients due to multiple factors. In addition, although the NLR is highly sensitive, its specificity is limited, and it has not been widely recognized as a definitive indicator of inflammation [25]. These confounding variables could explain the lack of significance in utilizing NLR to assess morbidity. Hence, future research endeavors should delve deeper into these factors and possibly adjust them to gain a more comprehensive understanding of the predictive capability of NLR concerning CRRT‐associated morbidities.

When assessing the predictive value of the NLR, it is important to consider the possible relationship between inflammation, malnutrition, and protein‐energy wasting [26]. Evaluating nutritional status can be done by examining lymphocytes and serum albumin levels. In addition, there is a significant relationship between the NLR and serum albumin levels [27]. Serum albumin has shown excellent predictive ability in patients receiving dialysis treatment. Additional research is needed to fully understand how this factor may impact the predictive value of the NLR to validate our findings and its potential clinical application. Therefore, it is necessary to conduct large‐scale, multicenter clinical trials due to the ongoing controversy surrounding the implications of NLR.

Our study highlights the potential of NLR as a valuable biomarker and indicator for the optimal timing of CRRT initiation in critically ill patients. Although the baseline NLR did not demonstrate statistical significance concerning mortality, there's notable evidence that patients who died had elevated NLR at baseline compared to those who survived during the 3‐day follow‐up post‐CRRT initiation. This suggests that early initiation of CRRT might offer advantages in identifying individuals who could benefit from timely intervention, potentially improving overall patient outcomes [28, 29]. These findings emphasize the significance of integrating NLR into clinical decision‐making algorithms for optimizing patient management and enhancing outcomes of critically ill patients on CRRT. Integrating NLR into clinical practice can lead to risk categorization, optimization of treatment strategies, and improved patient outcomes during CRRT. As previously discussed, obtaining an optimal NLR cutoff point might provide an objective indicator of the favorable CRRT initiation time in further research, which is needed to validate our findings and explore the mechanisms that govern the association between the NLR and critically ill patient prognosis in CRRT.

### 4.1 Limitations

Our study should acknowledge certain limitations, providing compelling evidence supporting NLR's prognostic value in CRRT patients. Our findings may be limited in generalizability due to the retrospective methodology of the study and the single‐center design. In addition, the observed associations between patient outcomes and NLR may be confounded by covariates such as comorbidities, drug interactions, and variations in CRRT protocols. Our findings need to be validated, and the clinical implications of NLR in critically ill patients on CRRT should be explored more thoroughly through future prospective studies with larger, multicenter cohorts.

### 4.2 Recommendations and Implications

The study recommends integrating NLR into clinical practice for critically ill patients on CRRT. Healthcare practitioners should use NLR measures in risk assessment processes to identify high‐risk patients and adopt early intervention strategies. Customized treatment strategies, determined by NLR levels and clinical indicators, can potentially enhance patient outcomes. Additional study is required to verify the predictive importance of NLR and its impact on clinical decision‐making. Collaboration and integrating multiple disciplines are essential for optimizing patient outcomes on CRRT.

## 5 Conclusion

Our study investigated the relationship between NLR and prognosis in critically ill patients on CRRT. Mortality was found to be significantly associated with elevated NLR levels. In addition, the NLR showed promise as an indicator of the optimal timing of CRRT initiation. These findings suggest that integrating NLR assessment into clinical practice could improve risk stratification and aid in treatment decision‐making for CRRT patients. Healthcare providers can potentially improve patient outcomes and the management of resources in ICU settings by incorporating NLR into routine evaluation protocols. While our study did not reveal any significant changes in morbidity concerning CRRT complications and length of hospitalization based on NLR, it sets the stage for further investigation. Additional research should be conducted to investigate and understand the underlying processes that interact with NLR and further the prognosis of critically ill patients on CRRT.

## Author Contributions

**Abdulqadir J. Nashwan:** conceptualization. **Abdulqadir J. Nashwan, Mutaz I. Othman, Dore C. Ananthegowda, Jayesh P. Janardhanan, Jamsheer P. Alikutty, Muftah A. Othman, Mohamad Y. Khatib, Ahmad A. Abujaber:** methodology, literature search, data collection, writing the draft and final review. **Kalpana Singh:** formal analysis. **Anas Ibraheem and Abdullah I. Hamad:** methods and critical review of the final manuscript. All authors have read and approved the final version of the manuscript.

## Ethics Statement

The Institutional Review Board (IRB) of the Medical Research Committee (MRC) at Hamad Medical Corporation (HMC) officially approved our study (MRC‐01‐23‐755).

## Consent

The authors have nothing to report.

## Conflicts of Interest

Abdulqadir J. Nashwan is an editorial board member of Health Science Reports and coauthor of this article. He is excluded from editorial decision‐making related to the acceptance of this article for publication in the journal. The other authors declare no conflicts of interest.

## Transparency Statement

The lead author, Abdulqadir J. Nashwan, affirms that this manuscript is an honest, accurate, and transparent account of the study being reported, that no important aspects of the study have been omitted, and that any discrepancies from the study as planned (and, if relevant, registered) have been explained.

## Data Availability

The data that support the findings of this study are available from the corresponding author upon reasonable request. The lead author [Mr. Abdulqadir Nashwan] had full access to all of the data in this study and takes complete responsibility for the integrity of the data and the accuracy of the data analysis.
